# Calcium current improves coincidence detection of the LIF model

**DOI:** 10.1186/1471-2202-15-S1-P86

**Published:** 2014-07-21

**Authors:** Yansong Chua, Moritz Helias, Abigail Morrison

**Affiliations:** 1 Institute of Neuroscience and Medicine (INM-6) and Institute for Advanced Simulation (IAS-6), Juelich Forschungszentrum, Juelich, Germany; 2 Bernstein Center Freiburg, Albert-Ludwigs University, Freiburg im Breisgau, Germany; 3 Institute for Cognitive Neuroscience, Faculty of Psychology, Ruhr University of Bochum, Bochum, Germany

## 

Dendritic spikes are known to improve efficacy of synaptic inputs in causing action potentials [1]. The calcium spike at distal apical dendrites of layer 5 pyramidal neurons has been observed in-vitro and argued to support the propagation of synaptic inputs from distal tufts to the soma [2]. When combined with a back-propagating action potential, a smaller distal current is sufficient to trigger a calcium spike [3]. Recently, it has also been shown in-vivo that dendritic spikes contribute to the neuronal activity [4,5].

Calcium spikes have been modeled in multi-compartment point neuron models using first order kinetics [6]. Here we show that calcium spikes, in the regime of large synchronous inputs on top of a background of weakly fluctuating synaptic noise can be well approximated by a threshold-triggered current of fixed waveform. The exact contribution of the calcium spike to the somatic membrane potential can then be analytically derived. Accurate predictions are only obtained if correlations between the membrane potential and synaptic conductances are taken into account [7].

Comparing neuron models with and without calcium dynamics, we find that the calcium current increases the sensitivity of the neuron's spiking response to sufficiently large coincident input. In numerical simulations carried out with NEST [8], we investigate the effect of the jitter of close to synchronous inputs on the probability to elicit a calcium spike. With increased jitter, fewer calcium spikes are elicited and their average amplitude decreases.
